# Safety, efficacy, and quality of life outcomes of pulsed field ablation in Japanese patients with atrial fibrillation: results from the PULSED AF trial

**DOI:** 10.1007/s10840-024-01912-w

**Published:** 2024-09-07

**Authors:** Teiichi Yamane, Tetsuo Sasano, Hirofumi Tomita, Daisetsu Aoyama, Shinsuke Miyazaki, Masateru Takigawa, Masaomi Kimura, Taihei Itoh, Seigo Yamashita, Jada M Selma, Jeffrey Cerkvenik, Atul Verma, Hiroshi Tada, on behalf of the PULSED AF Investigators

**Affiliations:** 1 Division of Cardiology, Department of Internal Medicine, The Jikei University School of Medicine, Tokyo, Japan; 2 Department of Cardiovascular Medicine, Tokyo Medical and Dental University, Tokyo, Japan; 3 Department of Cardiology, Hirosaki University Graduate School of Medicine, Hirosaki, Japan; 4 Department of Cardiovascular Medicine, Faculty of Medical Sciences, University of Fukui, Fukui, Japan; 5 Cardiac Ablation Solutions, Medtronic, Inc, Minneapolis, MN USA; 6 Division of Cardiology, McGill University Health Centre, Montreal, QC Canada

**Keywords:** Atrial fibrillation, Catheter ablation, Pulsed field ablation, Japan

## Abstract

**Background:**

Pulsed field ablation (PFA), a novel treatment for atrial fibrillation (AF), has yet to be evaluated in a Japanese cohort.

**Methods:**

In this sub-analysis of the PULSED AF trial, 12-month outcomes of paroxysmal AF (PAF) and persistent AF (PsAF) patients treated with PFA in four Japan centers were assessed. After a 90-day blanking period, primary efficacy was determined via freedom from a composite endpoint of acute procedural failure, arrhythmia recurrence, or antiarrhythmic drug escalation over 1 year. Patient improvement was evaluated via two quality of life (QoL) surveys (AFEQT and EQ-5D) at baseline and 12 months.

**Results:**

The analysis included 32 patients, 16 PAF and 16 PsAF, with PAF patients averaging 61.1 ± 10.6 years and PsAF patients averaging 62.8 ± 11.5 years of age. Females made up 31% of PAF and 25% of PsAF cohorts. Acute pulmonary vein isolation was achieved in 100% of both cohorts. The primary efficacy success rate at 12 months was 75.0% for PAF and 56.3% for PsAF patients. No primary safety events occurred. The mean AFEQT score significantly increased for both PAF (25.9 points, *p* < 0.0001) and PsAF (13.2 points, *p* = 0.0002) patients, while the EQ-5D-5L score improved significantly for PAF (0.12 points, *p* = 0.048) patients but not for PsAF (0.04 points, *p* = 0.08) patients.

**Conclusions:**

Similar to outcomes in the global cohort, ablation with the PulseSelect™ PFA catheter was efficient, effective, and safe in a Japanese population, resulting in improved QoL for PAF and PsAF patients.

**Clinical trial registration:**

ClinicalTrials.gov Identifier: NCT04198701

## Introduction

As a treatment for atrial fibrillation (AF), the goal of catheter ablation therapy is to create effective cardiac lesions without causing injury to adjacent structures or surrounding tissue [1, 2]. However, thermal energy sources are limited by the potential for causing collateral tissue damage [3]. Pulsed field ablation (PFA), a novel ablation technology, creates lesions non-thermally through the mechanism of irreversible electroporation (IRE) [4, 5]. IRE utilizes high electric field gradients to induce permanent cell membrane hyper-permeabilization. Unlike with thermal modalities, IRE induces cell death without imparting thermal energy, reducing potential for protein denaturation or damage to the tissue scaffold [6].

The PULSED AF trial results showed that PFA is effective in treating paroxysmal (PAF) and persistent (PsAF) AF, while not inducing thermally mediated complications [7]. In general, outcomes for AF patients vary worldwide; therefore, it is important to investigate outcomes of AF treatments for specific populations. There has been a myriad of population-specific subset analyses conducted within large clinical trials testing radiofrequency and/or cryoablation for AF [8–13]. Along with procedural efficiency, Japan patient-level outcomes have been evaluated for cryoballoon ablation (CBA) but have yet to be assessed for PFA [14–16]. Therefore, a sub-analysis of the PULSED AF trial was conducted to determine if PFA is safe, effective, and efficient in treating PAF and PsAF patients in Japan.

## Methods

### Trial design

The objective of the PULSED AF trial was to evaluate the safety and efficacy of treating patients with PAF and PsAF via a PFA system (PulseSelect™ Pulsed Field Ablation System, Medtronic, Minneapolis, MN). The global, prospective, multicenter, non-randomized, and single-arm design of the PULSED AF trial design has been previously described in detail [7]. An international steering committee supervised study design and execution, data analysis, and evidence dissemination. Following principles outlined in the Declaration of Helsinki, this sub-analysis was conducted to assess outcomes of PFA treatment in patients with PAF and PsAF in Japan. Each of the four centers in Japan received approval from their local ethics review boards.

### Study participants

Patients, ranging from 18 to 80 years old, who had failed at least one class I or class III antiarrhythmic drug (AAD) and who had recurrent symptomatic PAF or PsAF were included. A prior publication encompasses the comprehensive list of study inclusion and exclusion criteria [7]. This sub-analysis includes patients with PAF and PsAF that were enrolled and underwent pulmonary vein isolation (PVI) via PFA in Japan at the four participating centers. All patients provided written informed consent before enrolling in the study.

### Pulsed field ablation procedure

**Fig. 1:**
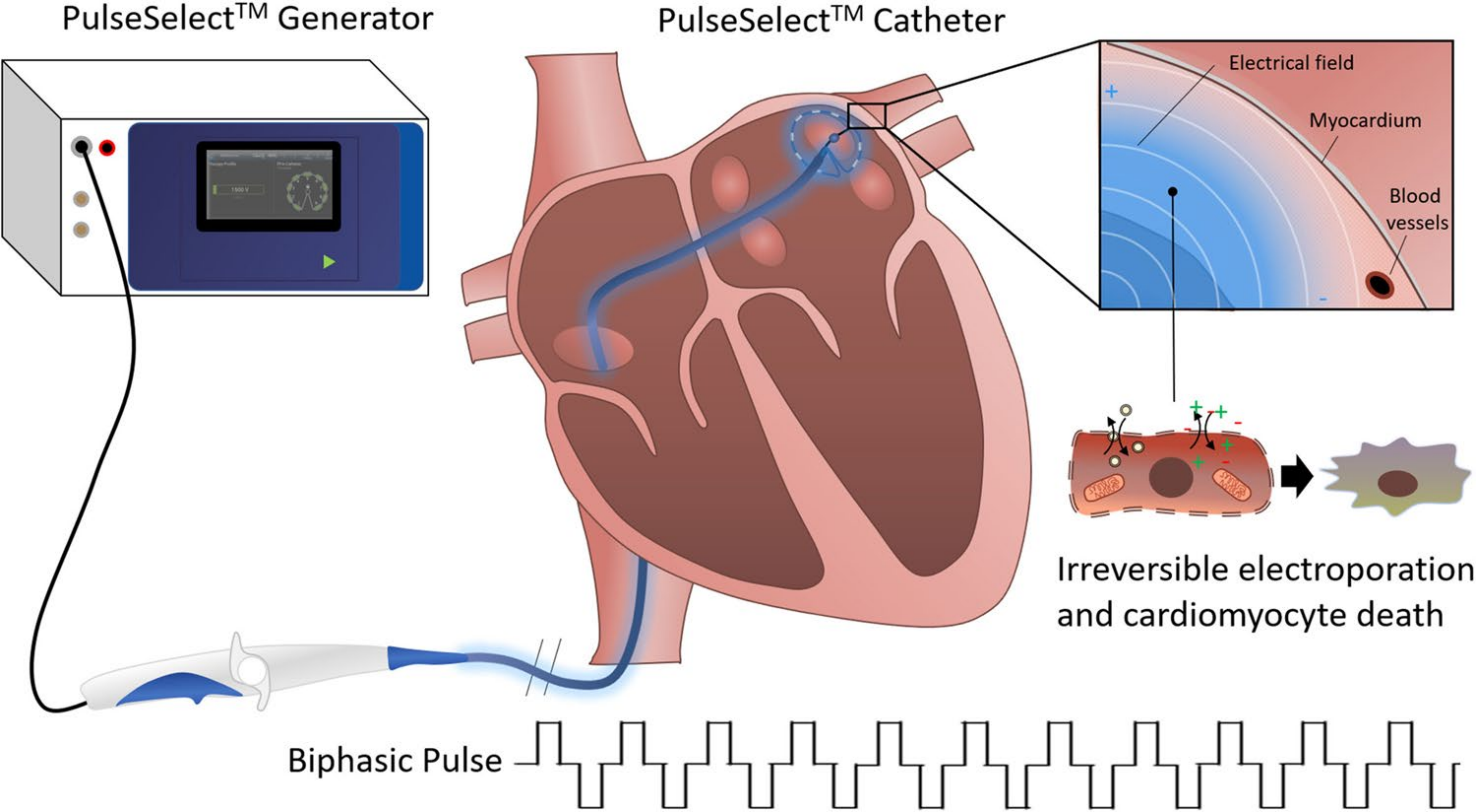
Pulmonary vein isolation using the PulseSelect^TM^ pulsed field ablation system. A biphasic pulse waveform is delivered via the generator, creating a bipolar electric field that is sustained around the electrode array of the catheter to ablate the cardiac tissue. PulseSelect’s waveform is optimized to induce irreversible electroporation in cardiomyocytes, leading to tissue specific cell death without disrupting the cellular matrix

A detailed description of the PFA procedure has been described in two previous PULSED AF publications [7, 17]. Briefly, clinicians introduced the PulseSelect catheter (9F) into the left atrium over a guidewire via transseptal puncture. A controlled biphasic, bipolar waveform was dispensed through a circular arrangement of 9 gold (3-mm-long electrodes) in the PulseSelect catheter. Fluoroscopy, intracardiac echocardiography (ICE) imaging, or mapping systems were employed to determine the positioning of the PFA catheter’s 25-mm circular array at every pulmonary vein (PV) (Figure 1). The PFA procedure did not require contrast media. One application was comprised of a series of four biphasic, bipolar pulse trains, each lasting 100–200 ms. After each application, the catheter was repositioned to achieve full circumferential isolation of the PV; a minimum of four ostial and four antral applications were delivered for each PV, as described previously. Following a mandatory 20-min wait period, confirmation of complete PVI via entrance block was required.

### Study follow-up and study endpoints

Study participants were monitored for 12 months via a combination of scheduled weekly and symptomatic transtelephonic monitoring (TTM), 12-lead ECGs (at 3, 6, and 12 months), and 24-h Holter monitoring (at 6 and 12 months). An independent core laboratory adjudicated arrhythmia monitoring events. Virtual or in-person visits at 1 week, 1, 3, and 12 months post-ablation were also conducted.

After the 90-day blanking period, efficacy was determined via freedom from a composite primary endpoint of acute procedural failure, arrhythmia recurrence of ≥ 30 s, repeat ablation, direct current cardioversion for atrial tachyarrhythmia recurrence, any subsequent left atrial surgery or ablation, or AAD escalation through 12 months. Within the blanking period, recurrent arrhythmias could be managed with AADs, cardioversion, or one repeat pulsed field ablation without being deemed a primary efficacy failure. Acute procedural failure was defined as the occurrence of any of the following: (1) inability to isolate all accessible, targeted PVs, which were minimally assessed for entrance block (and where assessable, exit block) and (2) ablation in the left atrium using a non-study device during the index procedure. AAD escalation included (1) prescribing of new class I or III AAD after the blanking period; (2) a class I or III AAD dose increase from the historic maximum ineffective dose after the blanking period; or (3) starting or using amiodarone at a dose greater than the maximum previous ineffective dose during the blanking period. Freedom from a composite of serious procedure- and device-related adverse events comprised the primary safety endpoint. The clinical events committee adjudicated whether each adverse event was associated with either the PulseSelect system or the PFA procedure.

The self-administered AF Effect on Quality of Life (AFEQT) Questionnaire, which is specific to AF health, and the European Quality of Life-5 Dimensions (EQ-5D) were utilized to assess QoL at baseline and 12 months. AFEQT scores range from 0 (complete AF-related disability) to 100 (no AF-related disability). The EQ-5D questionnaire has a composite score based on a five-question survey that ranges from 0 (least healthy) to 1 (most healthy). A clinically meaningful improvement in QoL is > 19 points for AFEQT and > 0.03 for EQ-5D [18, 19]. The study also collected episodes of healthcare utilization including hospitalizations, cardiovascular-related hospitalizations, emergency department visits, repeat ablations, and direct current cardioversions (12 months prior and post-ablation).

### Statistical analysis

In the text, continuous variables are presented as a mean and standard deviation and categorical variables are presented as a percentage. The primary effectiveness endpoint was evaluated using Kaplan–Meier analysis. QoL is reported as the change from baseline to 12 months and was evaluated using the paired students *t*-test. Statistical analyses were performed using SAS software Version 9.4 (SAS Institute, Cary, NC). *p*-values of < 0.05 were considered statistically significant.

## Results

### Patient and baseline characteristics

The study enrolled 421 patients, of which 44 were enrolled in Japan. Of these, 12 patients were not included in the primary endpoint analysis, with 4 not receiving an ablation and 8 stratified to a roll-in cohort. A total of 32 were in the pivotal phase of the study and received an ablation, including 16 paroxysmal AF (PAF) and 16 persistent AF (PsAF) patients. Baseline characteristics are displayed in Table 1. In both cohorts, the majority were male, encompassing 68.8% of the PAF and 75.0% the PsAF group. PAF and PsAF cohorts had an average age of 61.1 ± 10.6 and 62.8 ± 11.5 years, a mean body mass index of 24.5 ± 3.1 and 23.1 ± 2.7 kg/m^2^, and a CHA_2_DS_2_-VASc scores of 1.4 ± 1.7 and 1.5 ± 1.0, respectively.

**Table 1:** Baseline patient characteristics

Characteristic	Paroxysmal (*n* = 16)	Persistent (*n* = 16)
Male sex	11 (68.8)	12 (75.0)
Age (years)	61.1 ± 10.6	62.8 ± 11.5
Left atrial diameter (mm)	36.6 ± 4.8	39.7 ± 4.9
Left ventricular ejection fraction (%)	64.9 ± 5.0	60.6 ± 9.1
Body mass index (kg/m^2^)	24.5 ± 3.1	23.1 ± 2.7
Number of failed AADs		
1	14 (87.5)	15 (93.8)
2	2 (12.5)	1 (6.3)
Cardioversions within 12 months of enrollment		
Electrical (1)	0 (0.0)	1 (6.3)
Pharmacologic (1 or 2)	2 (12.5)	3 (18.8)
Medical characteristics		
Stroke	2 (12.5)	0 (0.0)
Transient ischemic attack	0 (0.0)	0 (0.0)
Myocardial infarction	0 (0.0)	0 (0.0)
Coronary artery disease	1 (6.3)	1 (6.3)
Hypertension	3 (18.8)	8 (50.0)
Obstructive sleep apnea	0 (0.0)	0 (0.0)
Valve dysfunction	0 (0.0)	0 (0.0)
Diabetes (type I)	1 (6.3)	0 (0.0)
CHA_2_DS_2_-VASc	1.4 ± 1.7	1.5 ± 1.0

Values are reported as mean ± SD or number (percentage)

### Procedural characteristics

Table 2 displays procedural characteristics. Including a protocol-mandated 20-min wait period and post-ablation mapping time, the median time between the first and last PFA application was 65.5 min for PAF and 51.5 min for PsAF patients. A total of 21 ± 2 and 22 ± 3 s of PFA energy were delivered per subject in the PAF and PsAF arm, respectively. All PFA procedures were performed with either conscious or deep sedation. In response to neuromuscular stimulation, each conscious sedation patient received additional sedative boluses during the procedure. Acute PV isolation was successfully achieved in 100% of 62 PVs in the PAF cohort and 64 PVs in the PsAF cohort. Due to a non-study device used to ablate left atrial anatomies (mitral valve isthmus) outside of the PVs, one acute procedural failure occurred in the PsAF group. ICE imaging was utilized in 44% of PAF patients and 38% of PsAF patients. All procedures utilized a mapping system to guide the positioning of the PFA catheter in each PV ostium/antrum (Table 2).

**Table 2:** Procedural characteristics

Parameter	Paroxysmal (*n* = 16)	Persistent (*n* = 16)
Skin-to-skin procedural time (min)^†^	146 ± 41 147.5 (111.5–175.5)	156 ± 63 133.5 (113.5–188.5)
Time between first and last application (min)^‡^	66 ± 27 65.5 (43–84)	64 ± 33 51.5 (47–78.5)
Fluoroscopy time during procedure (min)	45 ± 23 41 (24–65)	48 ± 28 47 (26–60)
Total pulsed field ablation energy delivered (sec)	21 ± 2 21 (19–22)	22 ± 3 23 (21–24)
Number of applications per procedure	40 ± 4 40 (37–43)	43 ± 5 43.5 (41–46.5)
Type of anesthesia used		
General anesthesia	0 (0)	0 (0)
Deep sedation	8 (50)	10 (63)
Conscious sedation	8 (50)	6 (38)
Neuromuscular blockade use	0 (0)	0 (0)
Isoproterenol and/or adenosine used to assess PVI	5 (31)	9 (56)
Intra-procedural cardioversions	4 (25)	12 (75)
Esophageal temperature change from baseline (°C)	0.3 ± 0.2^§^ 0.4 (0.3–0.4)	0.3 ± 0.1^¶^ 0.3 (0.2–0.4)
Intracardiac echocardiography	7 (44)	6 (38)
Mapping/navigation system used		
CARTO	2 (13)	2 (13)
EnSite	1 (6)	0 (0)
Rhythmia	12 (75)	11 (69)
None of the above	1 (6)	3 (19)
Acute Pulmonary Vein Isolation	62 (100)	64 (100)

Values are reported as mean ±SD, incidence number (percentage), or median (interquartile range)

^†^Skin to skin procedure time is from first sheath inserted to last sheath pulled out, including transfer time to recovery room and sheath pulling time

^‡^Time from first to last application includes the protocol-mandated 20-minute wait period and any post-ablation mapping time

^§^Data were available for 4 patients

^¶^Data were available for 2 patients

### Efficacy and safety

**Fig. 2:**
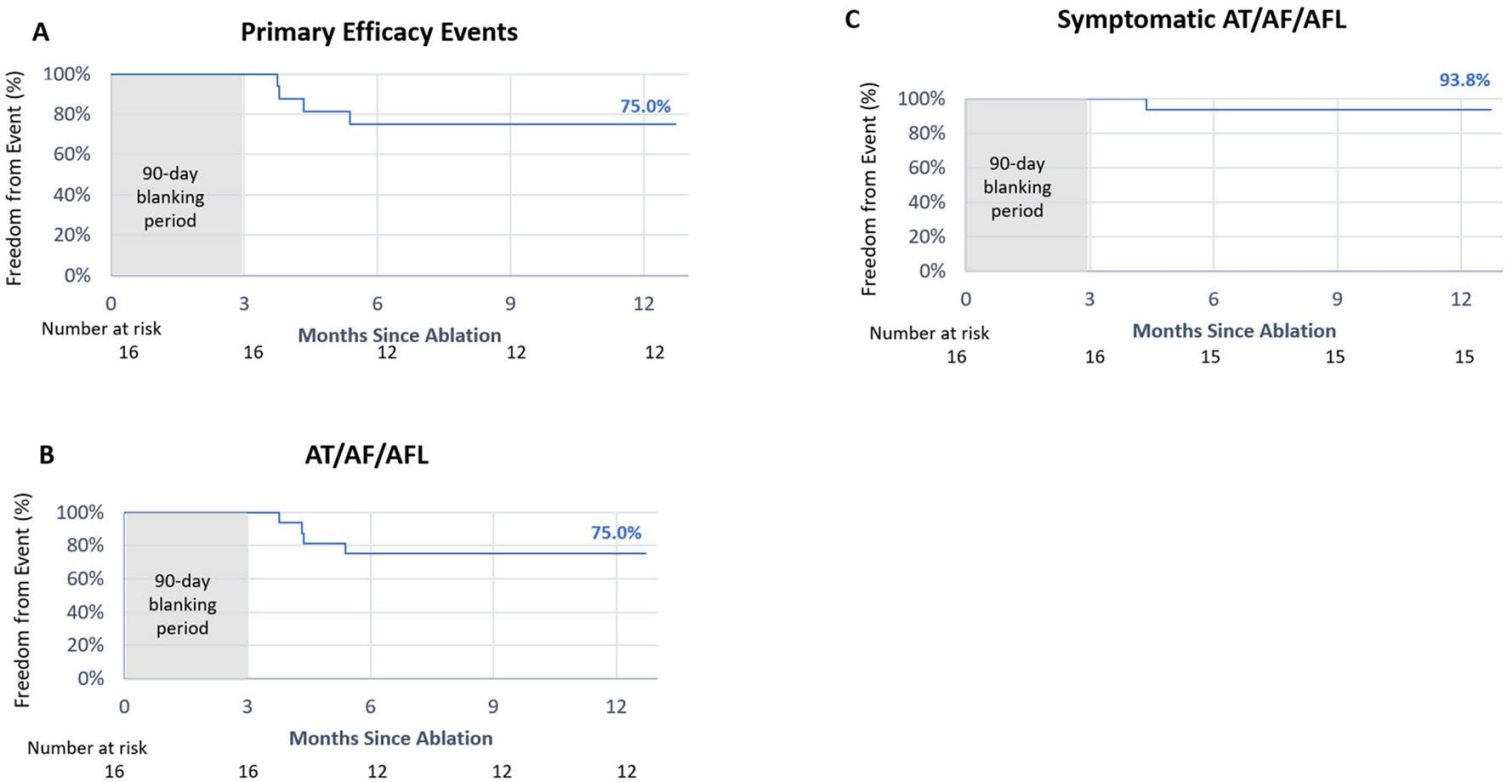
Treatment success at 12 months for paroxysmal atrial fibrillation. Kaplan–Meier estimate of freedom from **A** primary efficacy failure, **B** any atrial tachyarrhythmias (AF/AFL/AT) detected via Holter, ECG, or transtelephonic monitoring, and **C** atrial tachyarrhythmias detected via transtelephonic monitoring that occurred in conjunction with patient-reported symptoms at 12 months following a 90-day blanking period in patients with PAF. AF, atrial fibrillation; AFL, atrial flutter; AT, atrial tachycardia

**Fig. 3:**
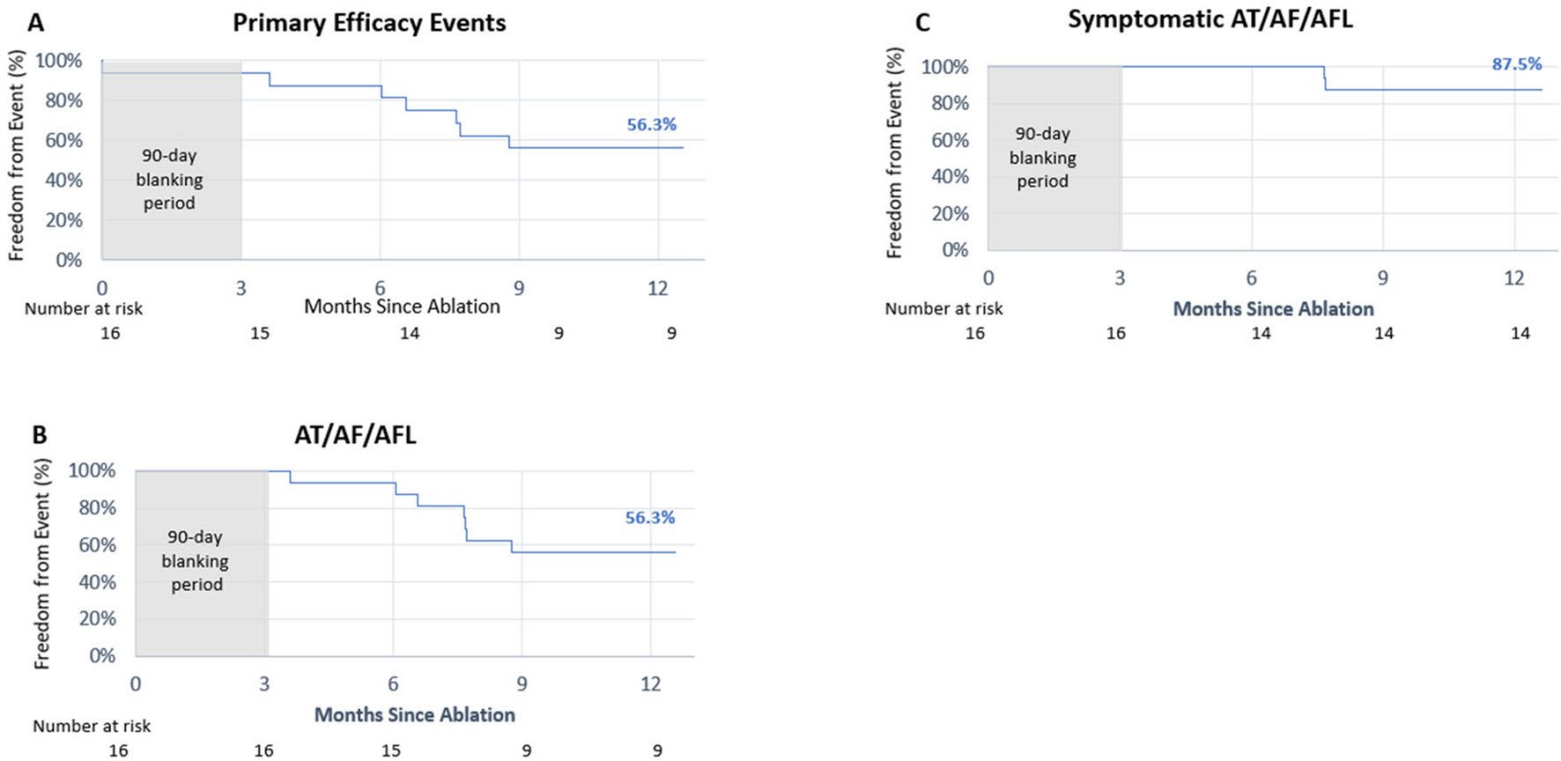
Treatment success at 12 months for persistent atrial fibrillation. Kaplan–Meier estimate of freedom from **A** primary efficacy failure, **B** any atrial tachyarrhythmias (AF/AFL/AT) detected via Holter, ECG, or transtelephonic monitoring, and **C** atrial tachyarrhythmias detected via transtelephonic monitoring that occurred in conjunction with patient-reported symptoms at 12 months following a 90-day blanking period in patients with PsAF. AF, atrial fibrillation; AFL, atrial flutter; AT, atrial tachycardia

All 32 patients were followed for 12 months and completed the trial through the 12-month visit. The Kaplan–Meier estimate of freedom from primary efficacy failure at 12 months was 75.0% for PAF patients and 56.3% for PsAF patients (Figs. 2A, 3A). The primary efficacy failure of documented arrythmia recurrence occurred in 18.8% of the PAF cohort and 37.5% of the PsAF cohort (Table 3). At 12 months, freedom from AF/atrial flutter (AFL)/atrial tachycardia (AT) was 75.0% for PAF patients and 56.3% for PsAF patients (Figs. 2B, 3B). Freedom from symptomatic AF/AFL/AT recurrence at 12 months was 93.8% and 87.5% in PAF and PsAF patients, respectively (Figs. 2C, 3C). The primary safety event rate was 0%, and no subsequent primary safety events occurred following repeat ablations.

**Table 3:** Primary effectiveness endpoint summary

Component	Mode of failure, *n* (%)	
	Paroxysmal (*n* = 16)	Persistent (*n* = 16)
Composite primary effectiveness failure	4 (25.0)	7 (43.8)
Individual components of effectiveness failure		
Acute procedural failure	0 (0.0)	1 (6.3)
Any subsequent atrial fibrillation surgery or ablation in the left atrium, except for one repeat pulmonary vein isolation ablation using pulsed field ablation within the 90-day blanking period	0 (0.0)	0 (0.0)
Direct current cardioversion for atrial tachyarrhythmia recurrences after the 90-day blanking period	0 (0.0)	0 (0.0)
Documented atrial fibrillation, atrial tachycardia, or atrial flutter on Holter/patient-activated ambulatory/12-lead ECG after the 90-day post-ablation blanking period	3 (18.8)	6 (37.5)
Atrial fibrillation	2 (12.5)	6 (37.5)
Atrial tachycardia	0 (0.0)	0 (0.0)
Atrial flutter	1 (6.3)	0 (0.0)
AAD use		
Dose increase from the historic maximum ineffective dose (prior to the ablation procedure) or initiation of a new class I or III AAD after the 90-day blanking period.	1 (6.3)	0 (0.0)

*AAD*, antiarrhythmic drug

### Healthcare utilization

Between ablation and 12 months, both study cohorts experienced the same rate of all-cause hospitalization (19%), cardiovascular-related hospitalization (13%), and emergency department visits (0%) (Table 4). Repeat ablation, which were all performed following a documented episode of AF with non-PFA systems and all were after the 90-day blanking period, occurred in 1 (6.3%) PAF patient and in 2 (12.5%) PsAF patients. While no PAF patients required direct current cardioversions, 6% of PsAF patients underwent this intervention during the 12-month follow-up.

**Table 4:** Healthcare utilization summary

Event between ablation and 12 months	Paroxysmal (*n* = 16) Number of subjects with event (%)	Persistent (*n* = 16) Number of subjects with event (%)
Hospitalization	3 (19)	3 (19)
CV hospitalization	2 (13)	2 (13)
Emergency department visit^†^	0 (0)	0 (0)
Repeat ablation	1 (6)	2 (13)
Direct current cardioversion	0 (0)	1 (6)

^†^Patients were included whether or not they were admitted to the hospital

### Quality of life

AFEQT and EQ-5D survey results at baseline and 12 months were acquired from all 32 patients. PAF patients experienced a significant as well as a clinically meaningful improvement in QoL according to both the AFEQT and EQ-5D scores. Improvements in QoL scores following PFA were not as pronounced in PsAF patients as compared PAF patients. For the AFEQT score, there was a significant mean increase of 25.9 (95% CI, 16.1–35.8, *p* < 0.0001) and 13.2 (95% CI, 7.3–19.0, *p* = 0.0002) point improvement from baseline to 12 months for PAF and PsAF patients, respectively (Table 5). At 12 months, there was a statistically significant 0.12-point (95% CI, 0.00–0.24, *p* = 0.048) improvement in the EQ-5D score in the PAF cohort; however, the 0.04-point (95% CI, -0.01–0.09, *p* = 0.08) improvement in the PsAF cohort did not reach statistical significance (Table 5). A clinically meaningful improvement occurred in both cohorts based on the EQ-5D results (> 0.03 points) but only in the PAF cohort based on the AFEQT results (> 19 points).

**Table 5:** Quality of life

Parameter	*n*	Baseline (mean ± SD)	12-month visit (mean ± SD)	Difference (95% CI)	*p*-value
Paroxysmal atrial fibrillation cohort					
Overall AFEQT Score	16	63.7 ± 21.9	89.6 ± 13.9	25.9 (16.1–35.8)	<0.0001
EQ-5D index value	16	0.83 ± 0.25	0.95 ± 0.09	0.12 (0.00–0.24)	0.048
Persistent atrial fibrillation cohort					
Overall AFEQT score	16	78.6 ± 13.6	91.8 ± 11.9	13.2 (7.3–19.0)	0.0002
EQ-5D index value	16	0.92 ± 0.19	0.96 ± 0.12	0.04 (− 0.01–0.09)	0.08

## Discussion

This study, to the best of our knowledge, is the first to evaluate PFA as a treatment for AF in a Japanese cohort. Excluding the protocol-mandated 20-min wait period, the time between the first and last PFA application averaged 46 ± 27 min for the PAF group and 44 ± 33 min for the PsAF group, with no more than a mean 23 s of energy being delivered to patients. Treatment success at 12 months occurred in 75% of PAF and 56.3% of PsAF patients in the absence of any serious procedure- and device-related adverse events. Both cohorts experienced a clinically meaningful improvement in EQ-5D QoL metrics. These results collectively demonstrate that PFA is an efficient, efficacious, and safe treatment that improves the QOL of PAF and PsAF patients in Japan.

The results of this sub-analysis of the PULSED AF trial are consistent with global outcomes within the PULSED AF Pivotal trial [7]. Similar to the global PULSED AF cohort, the median time between the first and last pulsed field application in the Japan cohort did not exceed 65 min for PAF and PsAF patients. Overall, a low duration (< 30 s) of pulsed field ablation energy were delivered per both AF cohorts in the Japan and global study populations. This procedural efficiency was achieved in the Japan cohort with no operators having any prior experience with the study device, demonstrating its ease-of-use and fast learning curve. While mapping systems were utilized in 95% of PAF and 92% of PsAF procedures in the global cohort, 100% of procedures in the Japan sub-study used an electroanatomical mapping system. Japanese operators did not utilize any general anesthesia, indicating that the PFA system can be used with a variety of standard-of-care practices, globally. By contrast, general anesthesia was the prevalent mode of sedation in the PULSED AF global cohort.

When comparing Japan results to the global cohort, the primary efficacy rate of PFA in PAF (75% vs 66.2%) and PsAF (56.3% vs 55.1%) patients were similar, respectively. Dissimilar to global results, freedom from AF/AFL/AT recurrence did not vary from the primary efficacy rate due to recurrence being the cause of most primary efficacy failures for both PAF and PsAF patients in the Japan cohort. No pre-specified primary safety events occurred in the Japanese cohort, while there were 2 primary safety events in the global cohort. Notably in PULSED AF patients outside of Japan, one primary event was adjudicated as device-related while the other was procedure-related in a cohort of 300 patients (0.7% primary safety event rate). Consequently, this data together demonstrate the safety of PFA procedures in totality (as this energy source is non-thermal by nature and selective for cardiomyocyte destruction) [7, 17, 20]. It should be noted that the global cohort results from Verma et al. do include the Japan cohort presented here [7]; however, the global cohort results do not greatly change when excluding patients enrolled in Japan (data not shown).

In brief, the procedural efficiencies, efficacy, and safety demonstrated in this Japan sub-study cohort of the PULSED AF trial are in alignment with the global results that were previously published [7]. Consequently, these data provide evidence of a uniform PFA response regardless of geographic practice, patient ancestry, and/or differences in standard-of-care procedures.

### Study limitations

This analysis has several limitations. The study was powered for the overall sample size, but not for the Japan subgroup; therefore, some of the results are skewed due to a small sample size. Data for esophageal temperature change was only available in 1/4 of PAF and 1/8 of PsAF patients; therefore, thermal effects to the esophagus may be underestimated. However, there were no evident signs of esophageal injury, including the absence of spams, dysphagia, or fistula.

## Conclusions

The PULSED AF trial results establishes PFA as an efficient, efficacious, and safe treatment for patients suffering from PAF and PsAF in Japan. QoL metrics improved in both groups following PFA, with larger improvements observed in PAF patients.

## Data Availability

Data will not be shared publicly because patient informed consent in the PULSED AF trial does not allow redistribution of patient data outside of the clinical trial usage.

## Abbreviations

AAD: Antiarrhythmic drug
AA: Atrial arrhythmia
AF: Atrial fibrillation
AFL: Atrial flutter
AT: Atrial tachycardia
CV: Cardiovascular
LA: Left atrium
PAF: Paroxysmal atrial fibrillation
PsAF: Persistent atrial fibrillation
PFA: Pulsed field ablation
PV: Pulmonary vein
PVI: Pulmonary vein isolation
